# 

**Published:** 1889-04-20

**Authors:** 


No. 134, Vol. VI.—April 20th, 1889.

[Registered at the General Post Office as a Newspaper.]

Monthly Parts, 6d.; post free, 7½d.

Ditto per Annum, 7s. 6d., post free.

